# Global comparison of breast cancer burden between women aged 20–54 and ≥55 years (1990–2021)

**DOI:** 10.3389/fonc.2025.1688642

**Published:** 2026-01-08

**Authors:** Xingxin Ouyang, Hao Liu, Huimin Jin

**Affiliations:** 1 Department of Breast Surgery, The Affiliated Cancer Hospital of Xiangya School of Medicine, Central South University/Hunan Cancer Hospital, Changsha, China; 2 Department of General Surgery, The Second Xiangya Hospital, Central South University, Changsha, China; 3 Department of Geriatric Surgery, The Second Xiangya Hospital, Central South University, Changsha, China; 4 Department of Hepatic Surgery, The Second Xiangya Hospital, Central South University, Changsha, China; 5 Department of Hepatobiliary Diseases, Hunan Provincial People’s Hospital, The First-Affiliated Hospital of Hunan Normal University, Changsha, China

**Keywords:** breast cancer, disease burden, EAPC, epidemiology, GBD database

## Abstract

**Background:**

While the global burden of breast cancer continues to rise, comparative long-term trends in incidence and mortality between reproductive-age and older women remain inadequately characterized across different socio-demographic settings.

**Methods:**

Data on female breast cancer incidence, mortality, and disability-adjusted life years (DALYs) from 1990 to 2021 were obtained from the Global Burden of Disease 2021 study. Trends were analyzed for two age groups (20–54 and ≥55 years). The Estimated Annual Percentage Change (EAPC) was calculated using joinpoint regression, which identifies significant trend changes by fitting a series of linear segments connected at “joinpoints.”Statistical comparisons of trends between age groups and SDI regions were conducted using t-tests based on the Joinpoint software framework.

**Results:**

Between 1990 and 2021, global breast cancer incidence increased in both age groups, with a more pronounced rise among women aged 20–54 years. Mortality and DALYs diverged: both increased in younger women but declined in those aged ≥55 years. Geographic disparities were strongly linked to SDI levels; although high-SDI regions reported higher incidence, they also showed steeper recent mortality reductions, highlighting the roles of risk factor profiles and healthcare access.

**Conclusion:**

This study offers a novel, age-stratified comparison of global breast cancer burden across SDI regions over three decades. Our findings underscore the necessity of age-specific and resource-stratified strategies for prevention and control to mitigate the growing burden in both younger and older women.

## Introduction

Breast cancer represents the most common malignancy and a leading cause of cancer-related mortality among women worldwide, imposing a substantial burden on global healthcare systems (1). Incidence and mortality rates vary considerably across regions, largely due to disparities in early screening, diagnostic capacity, and treatment accessibility (2, 3). For instance, incidence rates in developed countries are nearly 2.5 times higher than those in developing regions (4). Understanding these geographic disparities is essential for effective resource allocation, particularly in low- and middle-income countries.

The etiology of breast cancer involves multiple factors, including genetic predisposition (e.g., BRCA mutations, family history), breast density, reproductive history, breastfeeding duration, lifestyle factors (e.g., smoking, alcohol consumption, physical activity, and diet), and environmental exposures such as radiation (5). While non-modifiable factors play a significant role, modifiable lifestyle and environmental elements offer critical avenues for timely intervention. The Global Burden of Disease (GBD) 2021 study estimated the worldwide burden and risk-attributable impacts of breast cancer from 1990 to 2021 (6), and several studies have since utilized this database to analyze its epidemiology (7). However, the specific burden and trends of breast cancer among younger women remain inadequately characterized.

Breast cancer in younger women carries distinct physiological and psychosocial consequences, including impacts on sexual health, body image, mental well-being, and fertility, which affect not only individuals but also families and society (8–10). Given these age-specific implications, an updated and comprehensive assessment of the breast cancer burden across different age groups is urgently needed to support evidence-based policymaking and targeted healthcare planning.

The GBD study systematically quantifies the morbidity, prevalence, mortality, and health loss attributable to major diseases and injuries (11, 12). Using GBD 2021 data, this study estimates breast cancer incidence, mortality, and disability-adjusted life years (DALYs) from 1990 to 2021 across age groups, and at global, regional, and national levels, with stratification by Socio-demographic Index (SDI). These updated estimates reveal the spatiotemporal and geographical patterns of breast cancer burden, thereby providing scientific evidence to optimize preventive strategies and guide the prioritization of healthcare resources.

## Materials and methods

### Data source

The Institute for Health Metrics and Evaluation (IHME) leads the Global Burden of Disease (GBD) study, which systematically evaluates health loss attributable to diseases, injuries, and risk factors across 204 countries and regions. The 2021 GBD iteration continues this effort by providing comparative, quantifiable assessments of population health (12). Since its inception in 1990, the GBD study has employed standardized methodologies to generate annual estimates of disease burden, incorporating metrics such as incidence, prevalence, mortality, years of life lost (YLL), years lived with disability (YLD), and disability-adjusted life years (DALYs). DALYs combine YLL—years lost due to premature mortality—and YLD—years of healthy life lost due to disability—relative to a reference life expectancy. This composite measure offers a comprehensive quantification of disease impact, capturing both fatal and non-fatal health outcomes. Detailed methodological protocols for the GBD studies are accessible online.

Leveraging data from the 2021 GBD study, we analyzed female breast cancer (BC)-related morbidity, mortality, and DALYs across two age groups: 20–54 years and ≥55 years. This age stratification was necessitated, in part, by the structure of the GBD database, which reports data in standardized age groups rather than individual years. To ensure robust estimates and align with the GBD’s analytical framework, we consolidated the available 5-year age intervals into two broader cohorts that capture distinct life stages. This grouping effectively distinguishes the burden between primarily premenopausal women (20–54 years) and postmenopausal women (≥55 years), a approach consistent with prior epidemiological studies. Our analysis covers trends from 1990 to 2021, with stratification by age, region, and country. We extracted the data via the Global Health Data Exchange query tool, a resource developed through multinational collaboration and curated by the IHME.

### Socio-demographic index

The Institute for Health Metrics and Evaluation (IHME) introduced the Socio-demographic Index (SDI) in 2015 as a composite indicator to evaluate the developmental status of countries and regions, with a particular focus on the association between sociodemographic factors and population health. The SDI integrates three key metrics: the total fertility rate among women under 25, the average educational attainment in individuals aged 15 and older, and per capita income distribution. Within the GBD 2021 framework, 204 countries and regions are classified into five SDI tiers—low, low-middle, middle, high-middle, and high—to facilitate stratified analysis of health outcomes across different developmental contexts (13, 14).

### Definitions and measures

In the GBD, breast cancer (BC) is defined by the International Classification of Diseases, 10th edition (ICD-10). The primary aim of this study is to globally assess the overall disease burden of BC. We categorized the data into six regions defined by the World Health Organization (WHO): Africa, the Eastern Mediterranean, Europe, the Americas, Southeast Asia, and the Western Pacific. At the same time, countries and regions were classified based on SDI to analyze and compare the disease burden across different regions and levels of social development.

### Statistical analysis

The data extracted from GBD 2021 covers age-standardized rates (ASR) and their percentage changes from 1990 to 2021. The ASRs were calculated using the GBD 2021 standard population to allow for comparable comparisons across locations and over time. Prevalence, mortality, and DALYs are expressed as cases, deaths, and DALYs per 100,000 population. To assess the changes from 1990 to 2021, we calculated the rate of change using the following formula: (value in 2021 - value in 1990)/value in 1990.

To analyze the temporal trends in age-standardized rates, we performed a Joinpoint regression analysis. This method models trends using a series of connected linear segments, allowing for the identification of significant points where the trend changes direction (joinpoints). The analysis was conducted using the Joinpoint Regression Program (version 4.9.1.0; National Cancer Institute, Rockville, MD, USA). We set the maximum number of allowable joinpoints to 6 to permit a detailed characterization of trend fluctuations over the 32-year study period. The final model was selected based on the Bayesian Information Criterion (BIC), which balances model fit with complexity, and the overall goodness-of-fit was assessed using the coefficient of determination (R²).

For each segment identified by the model, we calculated the annual percentage change (APC). The overall trend for the entire period was summarized by the estimated annual percentage change (EAPC), which is a weighted average of the APCs. A trend was considered to have a significant increase if the EAPC and the lower bound of its 95% confidence interval (CI) were both > 0. Conversely, a trend was considered to have a significant decrease if the EAPC and the upper bound of its 95% CI were both < 0. Statistical comparisons of trends between age groups and SDI regions were conducted using t-tests based on the Joinpoint software framework, with a p-value of less than 0.05 considered statistically significant.

## Results

### BC burden among women aged 20–54 and 55+ years

Globally, there were 7,093,964 new breast cancer (BC) cases among women aged 20–54 years in 2021, compared with 13,210,977 cases among women aged 55 years and older. These figures accounted for 34.91% and 65% of the total global BC incidence, respectively. Between 1990 and 2021, the estimated annual percentage change (EAPC) in the incidence rate was 1.18% (95% UI: 1.12 to 1.24) in the younger group (20–54 years), versus 0.33% (95% UI: 0.30 to 0.36) in the older group (≥55 years), indicating a more rapid increase in incidence among younger women. This indicates a more rapid acceleration of the BC incidence burden among younger women globally, pointing to evolving risk factor exposures or diagnostic practices in this demographic.

In contrast, trends in BC-related adverse outcomes—mortality and disability-adjusted life years (DALYs)—diverged between age groups. While older women (≥55 years) showed declining trends, younger women (20–54 years) experienced increases over the same period. Specifically, the EAPC for mortality and DALYs in the older group were –0.44% (95% UI: –0.48 to –0.39) and –0.46% (95% UI: –0.50 to –0.43), respectively. In comparison, the younger group exhibited positive EAPCs of 0.14% (95% UI: 0.07 to 0.22) for both mortality and DALYs (**Table 1** and **Table 2**). These patterns suggest that although BC incidence is rising across all ages, notable progress has been made in reducing mortality and disability burden among older women, likely reflecting advances in treatment and survival in this population. This stark divergence suggests that advancements in early detection and systemic therapy over the past three decades have substantially improved outcomes for older women, yet have been insufficient to curb the rising burden of fatal and disabling outcomes in younger women, potentially reflecting differences in tumor biology, stage at diagnosis, and treatment efficacy between these age groups.

**Table 1:** Global distribution of breast cancer (BC) among women aged 20–54 years.

Measure		Prevalence(95%UI)			Mortality(95%UI)			DALYs(95%UI)		
		Case (95%UI)	ASR (95%UI)	EAPC (95%UI)	Deaths (95%UI)	ASR (95%UI)	EAPC (95%UI)	Years (95%UI)	ASR (95%UI)	EAPC (95%UI)
Global		7,093,964(6,650,309 to 7,622,593)	838.52 (805.71 to 870.77)	0.12 (1.12 to 1.24)	197,401 (183,486 to 211,359)	10.57 (9.82 to 11.31)	0.14 (0.07 to 0.22)	9,403,046 (8,752,327 to 10,089,739)	503.34 (468.50 to 540.09)	0.15 (0.07 to 0.22)
Region	Oceania	7,478 (5,971 to 9,529)	240.65 (192.17 to 396.67)	0.43 (0.27 to 0.59)	505 (393 to 663)	16.26 (12.64 to 21.34)	0.78 (0.67 to 0.89)	24,032 (18,645 to 31,844)	773.38 (600.02 to 1024.76)	0.73 (0.63 to 0.84)
	Asia	3,832,325(3,427,278 to 4,294,534)	340.93 (304.90 to 382.05)	2.74 (2.7 to 2.79)	115,866 (104,351 to 128,629)	10.31 (9.28 to 11.44)	0.97 (0.87 to 1.06)	5,501,,756 (4,930,956 to 6,103,119)	489.45(438.67 to 542.94)	0.94 (0.85 to 1.03)
	America	1,342,833(1,282,926 to 1,408,147)	533.25(9.46 to 559.19)	0.03 (-0.12 to 0.19)	27,665 (26,171 to 29,169)	10.99 (10.39 to 11.58)	-0.56 (-0.62 to -0.51)	1,331,564 (1,256,900 to 1,411,205)	528.73 (499.12 to 560.40)	-0.59 (-0.65 to -0.52)
	Africa	581,258(499,546 To 672,222)	198.49 (170.59 to 229.55)	2.16 (2.04 to 2.28)	30,496 (25,671 to 35,464)	10.41 (8.80 to 12.11)	1.1 (1 to 1.2)	1,449,512 (1,221,892 to 1,689,357)	494.99 (417.26 to 576.89)	1.09 (1 to 1.19)
	Europe	1,325,816(1,266,108 to 1,386,839)	672.71(642.41 to 703.67)	0.66 (0.5 to 0.81)	23,076 (21,822 to 24,468)	11.71 (11.07 to 12.41)	-1.37 (-1.48 to -1.26)	1,106,329 (1,040,802 to 1,177,656)	561.34 (528.10 to 597.53)	-1.28 (-1.39 to -1.17)
	Western Europe	818,673 (786,637 to 850,162)	838.52 (805.71 to 870.77)	0.51 (0.27 to 0.74)	11,024 (10,641 to 11,394)	11.29 (10.90 to 11.67)	-1.72 (-1.79 to -1.65)	538,192 (510,508 to 568,537)	551.24 (522.89 to 582.32)	-1.59 (-1.66 to -1.51)
	North America	653,467(625,264 to 683,451)	776.24 (742.74 to 811.86)	-0.58 (-0.84 to -0.31)	8,476 (8,125 to 8,841)	10.07 (9.65 to 10.50)	-1.74 (-1.86 to -1.61)	421,632 (397,130 to 446,122)	500.85 (471.74 to 529.94)	-1.71 (-1.83 to -1.59)
	Australasia	53,772 (47,510 to 60,372)	732.62(647.30 to 822.53)	0.37 (0.05 to 0.69)	700 (626 to 779)	9.54 (8.53 to 10.61)	-1.9 (-2.03 to -1.77)	34,712 (31,077 to 38,761)	472.94 (423.41 to 528.10)	-1.75 (-1.88 to -1.62)
	Central Latin America	308,799(263,433 to 352,246)	478.30(408.04 to 545.60)	2.79 (2.68 to 2.9)	6,576 (5,607 to 7,497)	10.19 (8.68 to 11.61)	1.14 (1.06 to 1.22)	315,960 (269,277 to 362,193)	489.40 (417.09 to 561.01)	1.11 (1.02 to 1.2)
	East Asia	1,624,475(1,257,125 to 2,066,960)	455.53(352.52 to 579.61)	3.77 (3.63 to 3.92)	30,370 (23,208 to 39,063)	8.52 (6.51 to 10.95)	0.48 (0.27 to 0.7)	1,448,845 (1,106,856 to 1,860,347)	406.28 (310.38 to 521.68)	0.46 (0.27 to 0.65)
Health System	Advanced Health System	2,447,364(2,371,213 to 2,529,272)	694.80 (673.18 to 718.05)	0.46 (0.28 to 0.65)	38,026 (36,583 to 39,527)	10.80 (10.39 to 11.22)	-1.28 (-1.4 to -1.17)	1,847,043 (1,764,218 to 1,943,979)	524.37 (500.86 to 551.89)	-1.2 (-1.31 to -1.1)
	Basic Health System	3,244,805(2,850,892 to 3,723,076)	417.87(367.14 to 479.46)	3.17 (3.09 to 3.24)	82,417 (73,378 to 92,700)	10.61 (9.44 to 11.94)	0.92 (0.81 to 1.02)	3,902,608 (3,476,117 to 4,412,737,709)	502.58(447.66 to 568.28)	0.88 (0.78 to 0.97)
	Limited Health System	1,313,885(1,181,030 to 1,459,510)	195.18 (175.44 to 216.81)	2.4 (2.22 to 2.57)	71,033 (63,551 to 79,493)	10.55 (9.44 to 11.81)	1.09 (0.96 to 1.22)	3,373,382 (3,017,621 to 3,773,739)	501.12 (448.27 to 560.59)	1.09 (0.98 to 1.21)
	Minimal Health System	81,544(64,732 to 98,227)	125.93(99.97 to 151.69)	1.37 (1.12 to 1.62)	5,732 (4,526 to 7,051)	8.85 (6.99 to 10.89)	0.62 (0.43 to 0.81)	279,960 (215,641 to 333,353)	418.44 (333.02 to 514.80)	0.66 (0.48 to 0.85)
Socio-demographic Index	High SDI	1,812,321(1,752,737 to 1,869,915)	722.40 (698.65 to 745.36)	0.28 (0.05 to 0.52)	25,116 (24,388 to 25,853)	10.01 (9.72 to 10.31)	-1.43 (-1.55 to -1.31)	1,234,762 (1,176,577 to 1,296,478)	492.18 (468.99 to 516.78)	-1.34 (-1.46 to -1.22)
	High-middle SDI	1,689,139(1,505,551 to 1,924,019)	529.24 (471.71 to 602.83)	1.79 (1.72 to 1.85)	33,681 (30,229 to 37,789)	10.55 (9.47 to 11.84)	-0.51 (-0.65 to -0.38)	1,603,976 (1,438,281 to 1,805,172)	502.55 (450.64 to 565.59)	-0.47 (-0.59 to -0.35)
	Middle SDI	2,248,739(2,040,023 to 2,480,368)	368.92 (334.68 to 406.92)	3.01 (2.96 to 3.06)	66,721 (60,198 to 73,784)	10.95 (9.88 to 12.10)	1.06 (0.99 to 1.12)	3,145,278 (2,839,380 to 3,467,582)	516.00 (465.82 to 568.88)	1.01 (0.94 to 1.07)
	Low-middle SDI	1,014,519(921,290 to 1,105,806)	221.43 (201.09 to 241.36)	2.65 (2.57 to 2.72)	50,655 (45,562 to 55,876)	11.06 (9.94 to 12.20)	1.43 (1.36 to 1.49)	2,404,029 (2,157,202 to 2,666,271)	524.71 (470.84 to 581.95)	1.41 (1.36 to 1.47)
	Low SDI	322,880(281,353 to 368,168)	141.03 (160.82 to 122.90)	1.69 (1.47 to 1.9)	21,036(18,171 to 24,108)	9.19 (7.94 to 10.53)	0.68 (0.52 to 0.85)	1,005,948 (868,517 to 1,155,471)	439.41 (379.38 to 504.73)	0.74 (0.58 to 0.89)

**Table 2:** Global distribution of breast cancer (BC) among women aged 55 years and older.

		Prevalence(95%UI)			Mortality(95%UI)			DALYs(95%UI)		
		Case (95%UI)	ASR (95%UI)	EAPC (95%UI)	Deaths (95%UI)	ASR (95%UI)	EAPC (95%UI)	Years (95%UI)	ASR (95%UI)	EAPC (95%UI)
Global		13,210,977(12,371,920 to 13,964,022)	1,679.79 (1,573.10 to 1,775.54)	0.33 (0.3 to 0.36)	462,902 (418,756 to 497,306)	58.86 (53.25 to 63.23)	-0.44 (-0.48 to -0.39)	10,805,143 (9,972,761 to 11,606,816)	1,373.88 (1,268.04 to 1,475.81)	-0.46 (-0.5 to -0.43)
Region	Oceania	4,800 (4,167 to 5,549)	816.63 (708.96 to 944.04)	0.32 (0.28 to 0.37)	444 (365 to 536)	75.49 (62.02 to 91.11)	0.51 (0.47 to 0.56)	11,770 (9,511 to 14,416)	2,002.21 (1,618.03 to 2,452.38)	0.51 (0.46 to 0.55)
	Asia	4,921,504(4,472,025 to 5,413,177)	1,081.15 (982.41 to 1,189.16)	2.06 (1.96 to 2.16)	190,210 (169,333 to 213,304)	41.79 (37.20 to 46.81)	0.66 (0.6 to 0.72)	4,802,,110 (4,281,098 to 5,398,746)	1,054.93(940.47 to 1,185.99)	0.58 (0.54 to 0.63)
	America	3,480,740(3,255,476 to 3,678,575)	2,783.50(2,603.36 to 2,941.71)	-0.74 (-0.79 to -0.69)	93,368 (83,396 to 99,296)	74.67 (66.69 to 79.41)	-1.2 (-1.26 to -1.15)	2,132,828 (1,957,757 to 2,270,683)	1,705.60(1,565.59 to 1,815.84)	-1.21 (-1.26 to -1.16)
	Africa	507,367(458,769 to 557,203)	902.83 (816.36 to 991.52)	2.04 (1.88 to 2.2)	40,864 (36,341 to 45,663)	72.71 (64.67 to 81.2 5 )	1.3 (1.26 to 1.34)	1,013,145 (893,553 to 1,141,655)	1,802.84 (1,590.03 to 2,031.58)	1.35 (1.3 to 1.4)
	Europe	4,272,271(4,016,742 to 4,521,765)	2,874.91(2,702.96 to 3,042.80)	0.78 (0.69 to 0.88)	137,299 (120,593 to 146,938)	92.39 (81.15 to 98.88)	-0.39 (-0.46 to -0.33)	2,832,128 (2,579,796 to 3,031,589)	1,905.81 (1,736.00 to 2,040.03)	-0.68 (-0.75 to -0.61)
	Western Europe	2,965,779 (2,759,139 to 3,139,085)	3,698.78 (3,441.07 to 3,914.92)	0.51 (0.4 to 0.63)	81,799 (68,973 to 88,720)	102.02 (86.02 to 110.65)	-0.87 (-0.91 to -0.82)	1,585,816 (1,393,195 to 1,722,586)	1,977.76 (1,737.53 to 2,148.33)	-1.2 (-1.24 to -1.17)
	North America	2,595,819(2,412,489 to 2,758,697)	4,308.46 (4,004.17 to 4,578.80)	-0.7 (-0.8 to -0.6)	50,465 (44,422 to 53,874)	83.76 (73.73 to 89.42)	-1.61 (-1.68 to -1.54)	1,134,179 (1,033,955 to 1,221,044)	1,882.48 (1,716.13 to 2,026.65)	-1.62 (-1.66 to -1.57)
	Australasia	168,142 (153,730 to 181,694)	3,629.28(3,318.21 to 3,921.79)	0.26 (0.16 to 0.37)	3,619 (3,013 to 4,152)	78.12 (65.05 to 89.64)	-1.35 (-1.42 to -1.29)	77,530 (67,705 to 86,896)	1,673.46 (1,461.39 to 1,875.61)	-1.49 (-1.54 to -1.43)
	Central Latin America	342,603(307,481 to 378,412)	1,482.56(1,330.58 to 1,637.52)	2.07 (2.02 to 2.12)	12,743 (11,194 to 14,261)	55.14 (48.44 to 61.71)	0.56 (0.46 to 0.67)	314,946 (278,013 to 354,588)	1,362.88 (1,203.06 to 1,534.42)	0.63 (0.53 to 0.73)
	East Asia	2,275,067(1,907,231 to 2,689,150)	1,189(942.17 to 1,328.44)	2.6 (2.45 to 2.74)	62,568 (49,875 to 77,057)	30.91 (24.64 to 38.07)	-0.14 (-0.25 to -0.02)	1,626,668 (1,294,665 to 2,014,135)	803.58 (639.57 to 994.98)	-0.05 (-0.13 to 0.03)
Health System	Advanced Health System	8,021,807(7,487,264 to 8,473,853)	3,102.94 (2,896.15 to 3,277.78)	0.44 (0.38 to 0.5)	211,018 (184,402 to 225,127)	81.62 (71.33 to 87.08)	-0.66 (-0.71 to -0.62)	4,472,432 (4,038,428 to 4,775,629)	1,729.98 (1,562.11 to 1,847.26)	-0.87 (-0.91 to -0.83)
	Basic Health System	4,047,851(3,668,210 to 4,527,015)	1,168.71(1,059.10 to 1,307.06)	2.12 (2.03 to 2.21)	153,912 (137,564 to 171,754)	44.44 (39.72 to 49.59)	0.23 (0.14 to 0.31)	3,872,391 (3,454,163 to 4,334,223)	1,118.05(997.30 to 1,251.39)	0.26 (0.21 to 0.31)
	Limited Health System	1,069,462(971,322 to 1,173,417)	627.65 (570.05 to 668.66)	1.72 (1.57 to 1.88)	90,994 (80,540 to 101,155)	53.40 (47.27 to 59.37)	1.09 (1 to 1.19)	2,287,737 (2,019,210 to 2,563,74 36)	1,342.64 (1,185.04 to 1,504.44)	0.98 (0.86 to 1.1)
	Minimal Health System	58,066(49,739 to 67,259)	553.02(471.71 to 640.57)	1.03 (0.86 to 1.2)	6,358 (5,160 to 7,640)	60.55 (49.15 to 72.76)	0.91 (0.82 to 1)	159,106 (127,551 to 193,019)	1,515.32 (1,214.79 to 1,838.31)	0.82 (0.71 to 0.94)
Socio-demographic Index	High SDI	6,228,039(5,799,595 to 6,594,534)	3,377.27 (3,144.94 to 3,576.00)	0.17 (0.12 to 0.23)	146,332 (126,691 to 156,708)	79.35 (68.70 to 84.98)	-1.04 (-1.09 to -0.98)	3,087,353 (2,772,697 to 3,314,519)	1,674.17 (1,503.55 to 1,797.36)	-1.17 (-1.19 to -1.14)
	High-middle SDI	3,351,584(3,097,115 to 3,627,031)	1,783.68 (1,648.26 to 1,930.27)	0.87 (0.84 to 0.91)	112,229 (100,366 to 121,828)	59.73 (53.41 to 64.84)	-0.44 (-0.57 to -0.3)	2,584,177 (2,354,517 to 2,834,938)	1,375.28 (1,253.05 to 1,508.73)	-0.6 (-0.7 to -0.5)
	Middle SDI	2,527,197(2,308,029 to 2,748,032)	1,026.82 (937.77 to 1,116.54)	1.96 (1.87 to 2.04)	114,635 (102,529 to 127,678)	46.58 (41.66 to 51.88)	0.42 (0.36 to 0.49)	2,887,091 (2,582,370 to 3,234,955)	1,173.04 (1,049.23 to 1,314.38)	0.4 (0.34 to 0.46)
	Low-middle SDI	859,225(791,316 to 930,582)	684.67 (630.55 to 741.53)	2 (1.87 to 2.13)	64,873 (57,552 to 71,575)	51.69 (45.86 to 57.03)	1.39 (1.35 to 1.43)	1,635,140 (1,452,975 to 1,809,337)	1,302.95 (1,157.79 to 1,441.76)	1.3 (1.25 to 1.36)
	Low SDI	231,204(208,848 to 253,147)	552.61 (499.17 to 605.04)	1.2 (1.02 to 1.38)	24,212(21,517 to 27,223)	57.87 (51.43 to 65.07)	0.94 (0.83 to 1.04)	597,904 (526,540 to 674,723)	1,429.07 (1,258.50 to 1,612.68)	0.8 (0.68 to 0.92)

### Temporal trends in BC burden across regions between women aged 20–54 and55+ years

We examined the burden of breast cancer (BC)—measured by prevalence, mortality, and disability-adjusted life years (DALYs)—among women aged 20–54 years and those aged 55 years and older across World Health Organization (WHO) regions from 1990 to 2021.

**Figure 1:**
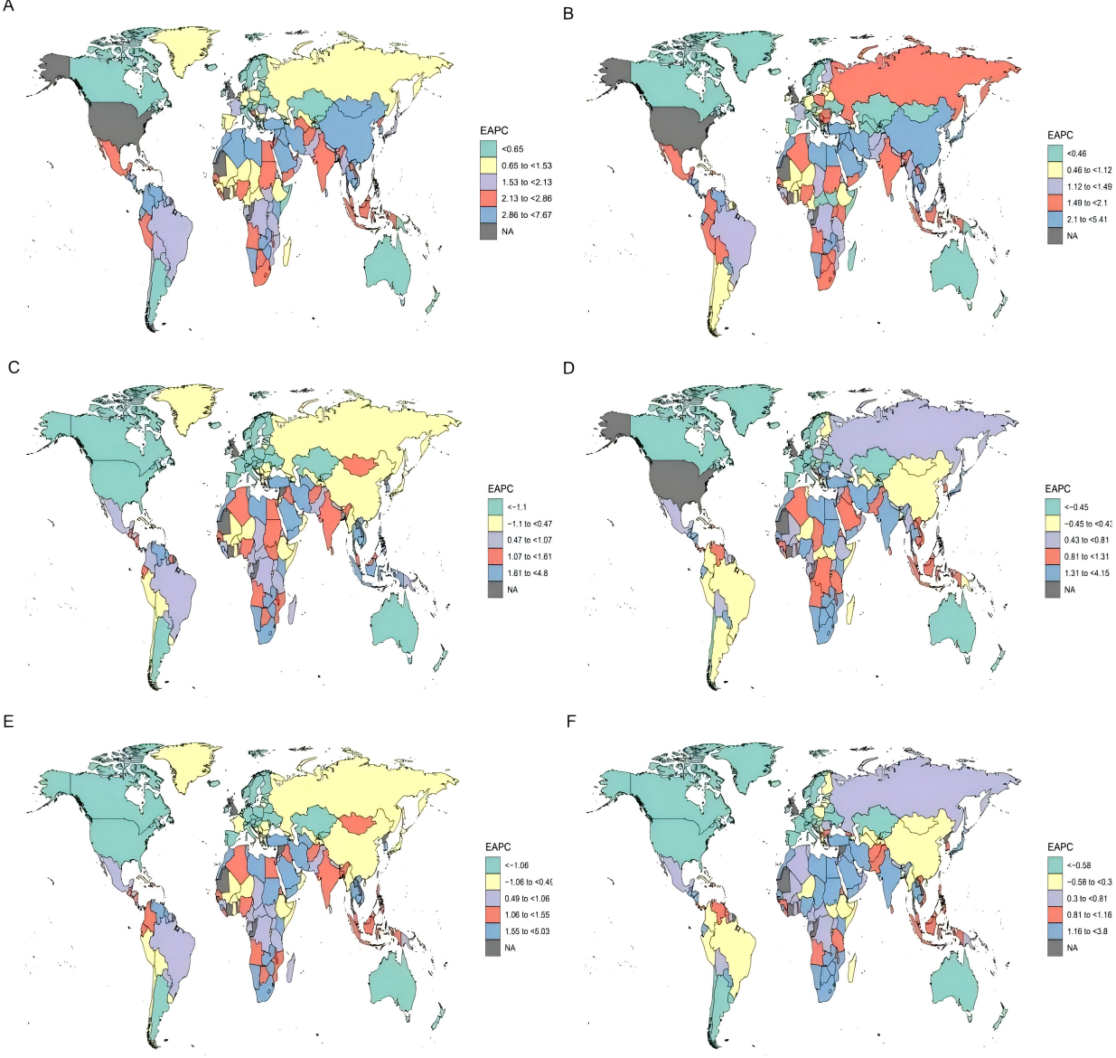
Global prevalence of BC: a world map overview. **(A)** Global Prevalence of BC among women Aged 20–54 Years. **(B)** Global Prevalence of BC among women Aged 55 Years and Older. **(C)** Global Deaths of BC among women Aged 20–54 Years. **(D)** Global Deaths of BC among women Aged 55 Years and Older. **(E)** Global DALYs of BC among women Aged 20–54 Years. **(F)** Global DALYs of BC among women Aged 55 Years and Older.

Among women aged 20–54 years, the North Africa and Middle East region experienced the largest increase in BC prevalence, with an estimated annual percentage change (EAPC) of 4.04% (95% UI: 3.87 to 4.21). In the same age group, Sub-Saharan Africa saw the greatest rise in BC mortality (EAPC 2.1%; 95% UI: 1.68 to 2.53), while North Africa and the Middle East recorded the highest increase in DALYs (EAPC 2.09%; 95% UI: 1.95 to 2.23).The steep increases in incidence and adverse outcomes in these regions likely reflect the combined effects of rapidly changing reproductive and lifestyle patterns, rising obesity, and healthcare systems that are still developing capacity for timely diagnosis and effective treatment. By contrast, South America showed the most pronounced declines in BC prevalence, mortality, and DALYs among young women worldwide (**Table 1** and **Figures 1A, C, E**).

Among women aged 55 years and above, the North Africa and Middle East region also exhibited the highest increase in BC prevalence, with an EAPC of 3.66% (95% UI: 3.33 to 4.0). Similarly, this region demonstrated the greatest increases in both mortality and DALYs among older women, with corresponding EAPCs of 2.51% (95% UI: 2.2 to 2.83) and 2.51% (95% UI: 2.21 to 2.81). In contrast, the Americas region overall showed the most substantial decline in BC prevalence (EAPC –0.74%; 95% UI: –0.79 to –0.69), whereas South America displayed the steepest reductions in BC mortality and DALYs globally in this age group, with EAPCs of –1.61% (95% UI: –1.68 to –1.54) and –1.62% (95% UI: –1.66 to –1.57), respectively (**Table 2** and **Figures 1B, D, F**). The notable progress in the Americas, particularly South America, highlights the potential for effective BC control in older women through improved healthcare access and adoption of effective therapies, even in settings that are not the most resource-abundant.

### SDI-related BT burden for women aged 20–54 and 55+ years across locations in 2021

**Figure 2:**
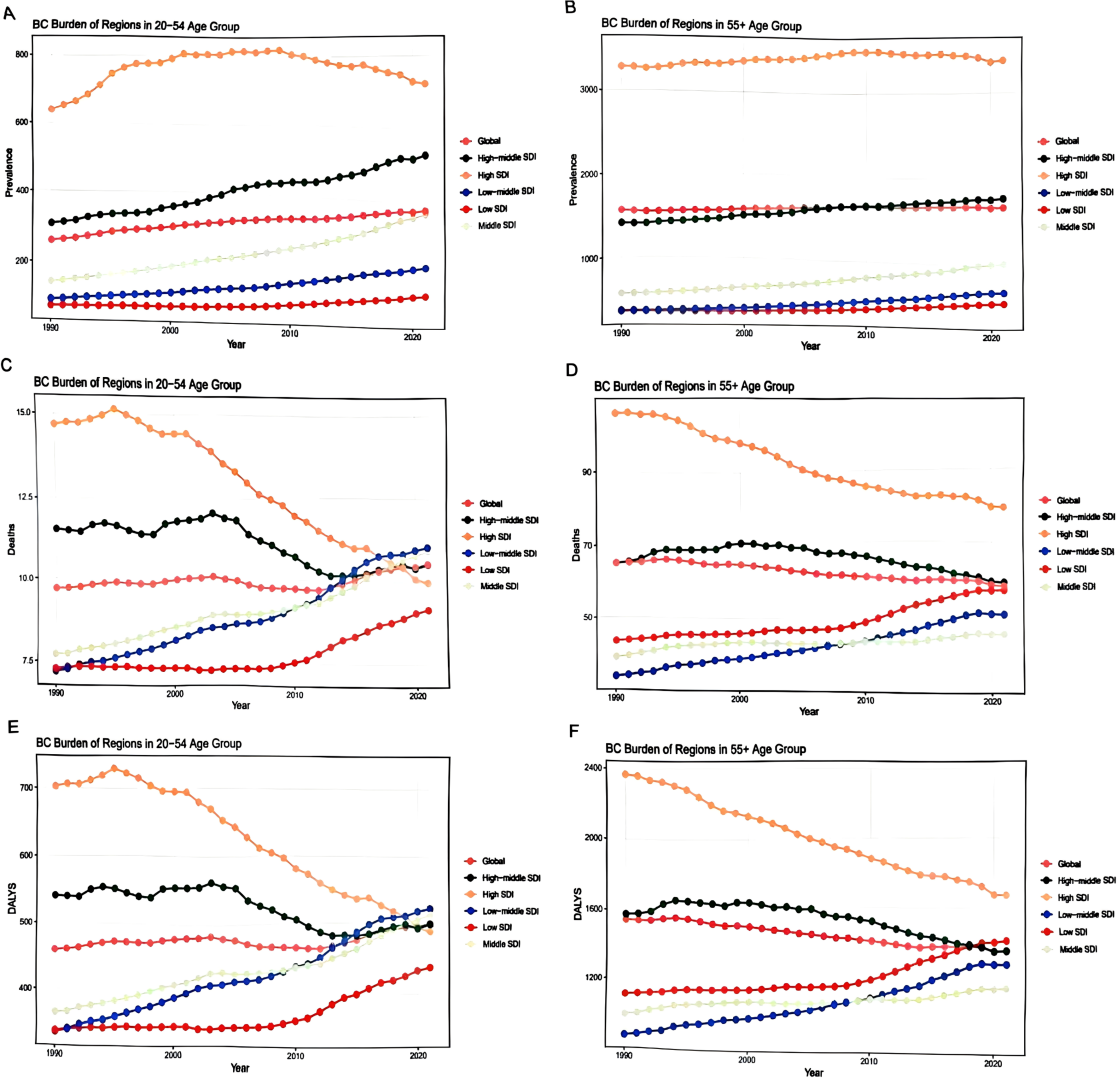
Changes in BC burden rates across SDI regions (1990-2021). **(A)** Prevalence of BC among women Aged 20–54 Years (1990-2021). **(B)** Prevalence of BC among women Aged 55 Years and Older (1990-2021). **(C)** Mortality Rate of BC among women Aged 20–54 Years (1990-2021). **(D)** Mortality Rate of BC among women Aged 55 Years and Older (1990-2021). **(E)** Disability-Adjusted Life Years (DALYs) for BC among women Aged 20–54 Years (1990-2021). **(F)** Disability-Adjusted Life Years (DALYs) for BC among women Aged 55 Years and Older (1990-2021).

Breast cancer (BC) trends among women varied across age groups and Socio-demographic Index (SDI) regions. Between 1990 and 2021, BC prevalence in women aged 20–54 years exhibited a positive correlation with SDI levels. Women in high and upper-middle SDI regions demonstrated significantly higher BC prevalence compared to the global average, whereas those in middle, lower-middle, and low SDI regions showed lower-than-global rates. This pattern likely reflects a combination of higher exposure to established risk factors and more intensive screening practices in these regions. Over this period, BC prevalence increased across all five SDI regions, with the most rapid growth observed in the middle SDI group, where rates among younger women have now approached the global average (**Figure 2A**).This suggests an ongoing epidemiological transition, where middle-income populations are experiencing a rapid escalation of BC incidence as they adopt Westernized lifestyles.

Regarding mortality, female BC deaths in high and upper-middle SDI regions displayed a declining trend, with the most pronounced reduction in high SDI areas, where mortality rates became significantly lower than those in upper-middle SDI regions. In contrast, BC mortality in middle, lower-middle, and low SDI regions, as well as globally, increased from 1990 to 2021. By 2021, mortality in the middle and lower-middle SDI regions had surpassed the global average, although it remained lower in the low SDI region (**Figure 2C**). Trends in disability-adjusted life years (DALYs) closely mirrored mortality patterns, with the most substantial decline again observed in high SDI regions (**Figure 2E**). This divergence underscores a critical gap in outcomes: high-SDI regions have successfully leveraged healthcare advancements to reduce mortality, while many lower-resource regions face rising mortality despite lower incidence, likely due to later-stage diagnosis and limited access to quality treatment.

Among women aged 55 years and older, BC prevalence in high SDI regions was markedly elevated compared to other SDI categories and the global level, while rates in upper-middle SDI regions aligned closely with global estimates. All five SDI regions, along with the global aggregate, experienced rising BC prevalence from 1990 to 2021, with the most notable increases occurring in upper-middle and middle SDI regions (**Figure 2B**). In terms of mortality and DALYs, both high and upper-middle SDI regions, together with the global average, exhibited declining trends, with the greatest reductions seen in high SDI settings. Conversely, middle, lower-middle, and low SDI regions experienced increases in BC mortality and DALYs. Throughout this period, the low SDI region consistently reported higher mortality and DALY rates than the middle and lower-middle SDI regions (**Figures 2D, F**).The sustained high burden in low-SDI settings highlights the profound challenges faced by older women in the most resource-constrained environments, where barriers to timely diagnosis, curative therapy, and palliative care are most severe.

### Joinpoint regression analysis

**Figure 3:**
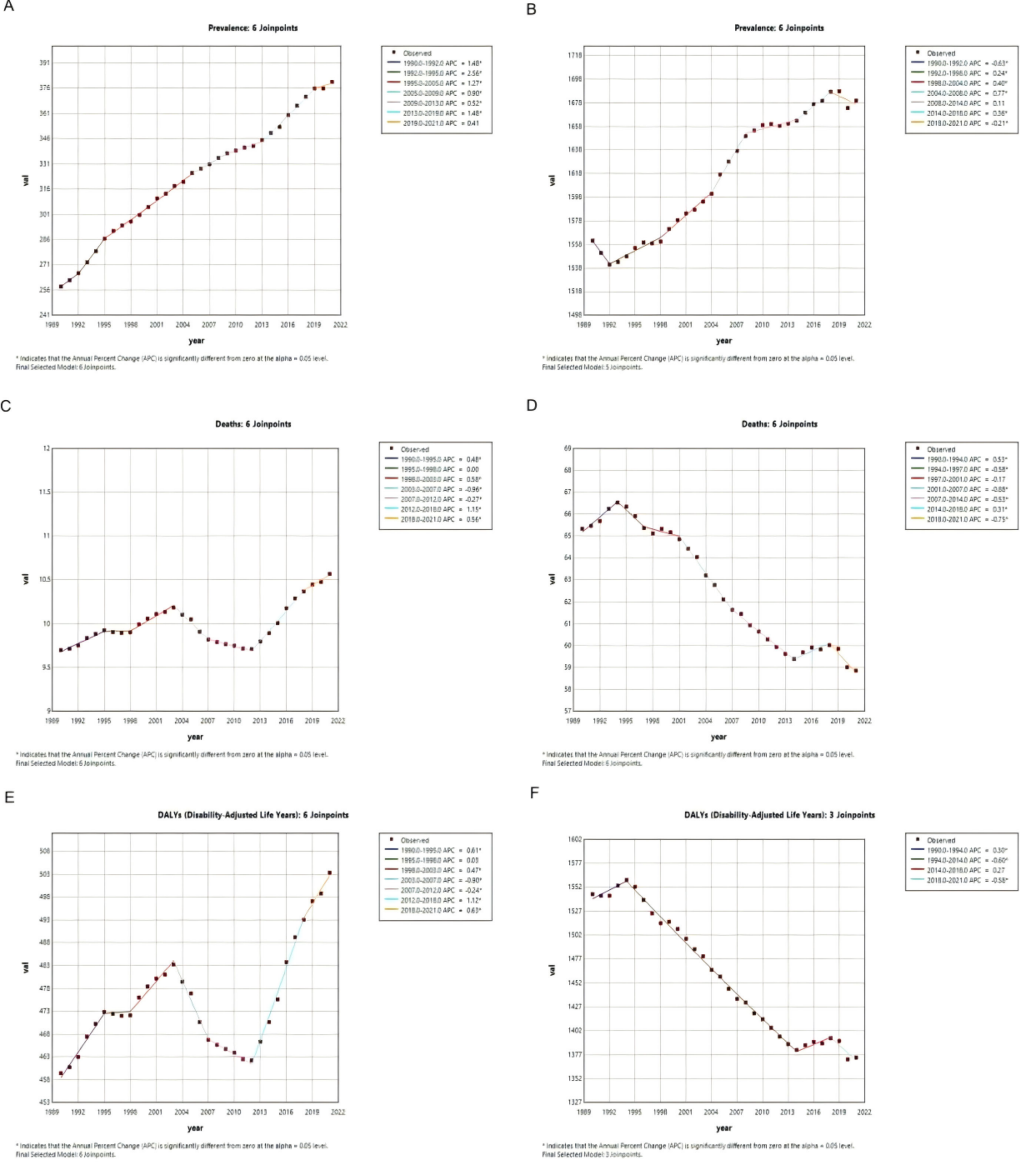
Joinpoint regression analysis of burden rates of BC across age groups (20–54 years *vs*. 55+ years). **(A)** The Joinpoint regression analysis of prevalence among women aged 20–54 years. **(B)** The Joinpoint regression analysis of prevalence among women aged 55 years and older. **(C)** The Joinpoint regression analysis of Deaths among women aged 20–54 years. **(D)** The Joinpoint regression analysis of Deaths among women aged 55 years and older. **(E)** The Joinpoint regression analysis of DALYs among women aged 20–54 years. **(F)** The Joinpoint regression analysis of DALYs among women aged 55 years and older.

We performed Joinpoint regression analysis to evaluate trends in the prevalence, mortality, and disability-adjusted life years (DALYs) of breast cancer (BC) among women aged 20–54 years and those 55 years and older worldwide; the results are presented in **Figure 3**.

Among women aged 20–54 years, global BC prevalence exhibited a sustained increase from 1990 to 2021. The most pronounced upward trends occurred during 1992–1995 and 2013–2019, with annual percentage changes (APCs) of +2.56% and +1.48%, respectively (**Figure 3A**). Similarly, BC mortality and DALYs in this age group also showed an overall increase over the entire period, despite a noticeable decline between 2003 and 2012 (**Figures 3C, E**). This temporary interruption in the upward trend might reflect the initial rollout of certain therapeutic advances or public health initiatives, the effects of which were not sustained in the face of rising incidence or more aggressive tumor subtypes in younger women.

For women aged 55 years and older, global BC prevalence generally rose from 1990 to 2021, with the most substantial increase observed between 2004 and 2008 (APC = +0.77%). However, a recent decline was noted from 2018 to 2021, with an APC of –0.21% (**Figure 3B**). In contrast, both BC mortality and DALYs in this older cohort demonstrated an overall downward trend across the study period (**Figures 3D, F**). The persistent decline in mortality and DALYs reinforces the conclusion that therapeutic and diagnostic strategies have been particularly effective in reducing the fatal impact of breast cancer in postmenopausal women.

## Discussion

Our analysis reveals a critical divergence in the global breast cancer epidemic: incidence is rising more rapidly among women aged 20–54, while mortality is declining only in those aged ≥55. This age-based disparity, starkly evident across the SDI spectrum, underscores the need for demographically tailored and resource-stratified control strategies.

The rising incidence, particularly among younger women, signals a shift in population-level risk profiles. The global trends toward delayed childbirth, lower parity, and reduced breastfeeding duration, compounded by rising obesity and physical inactivity, are established drivers (15–17). These factors, increasingly prevalent in transitioning economies, present a pivotal opportunity for primary prevention. Public health campaigns promoting healthy weight, physical activity, and reproductive health are essential to curb future incidence, especially in low- and middle-income countries (LMICs) where these risk factors are emerging most rapidly (18).

The mortality divide, however, primarily reflects inequitable access to and benefit from medical advances. The sustained decline in mortality among older women is a direct success of systematic mammography screening and the widespread adoption of highly effective adjuvant therapies, such as aromatase inhibitors and trastuzumab, in high-resource settings (19–21). These interventions are particularly effective against the hormone receptor-positive and HER2-positive subtypes that dominate in this age group. In stark contrast, younger women are more frequently diagnosed with aggressive subtypes like triple-negative breast cancer and face diagnostic delays, limiting the impact of these same advances and leading to stable or rising mortality (22, 23). This biological and clinical heterogeneity necessitates distinct approaches.

These findings necessitate a strategic reorientation of global breast cancer control toward LMICs, integrating age-specific priorities and health system strengthening. For younger women, resource allocation should shift toward scalable early detection—such as clinical breast examination coupled with rapid diagnostic pathways—rather than resource-intensive mammography screening, which demonstrates limited effectiveness in this group (24), while primary prevention efforts must concurrently address the growing burden of modifiable risk factors. For older women, improving access to affordable and effective treatment is critical; progress observed in parts of South America illustrates that outcomes can be enhanced through the integration of generic drugs and biosimilars into public health systems, thereby expanding the availability of essential therapies. Underpinning these approaches is the fundamental strengthening of health systems across LMICs to support timely diagnosis, reliable pathology services, and affordable treatment, ensuring that early detection ultimately translates into lives saved (25, 26).

This study has several limitations. First, our analysis relies on modeled estimates from the GBD study, which are subject to methodological constraints and inherent uncertainties. The accuracy of GBD estimates depends heavily on the quality and coverage of underlying cancer registration data, which are often incomplete or outdated in many low- and middle-income regions (27). In such data-sparse settings, the models employ predictive covariates and spatial smoothing, which may obscure local heterogeneity or introduce bias where empirical data are lacking. Furthermore, the GBD framework does not readily allow for the exclusion of data from countries with particularly low reliability, meaning that analyses may inevitably incorporate estimates from settings with very weak underlying data, potentially affecting the overall robustness of regional and global trends.

Second, although GBD performs extensive internal validation, external validation of breast cancer estimates—particularly in low-SDI settings—remains limited. Comparisons with registry data from Nigeria and Uganda revealed considerable discrepancies, suggesting that reported incidence increases in some low-income regions may still be underestimated and should be interpreted cautiously. Third, the absence of molecular subtype data (e.g., hormone receptor status) represents a critical limitation. The rising incidence in younger women and declining mortality in older women may reflect distinct subtype distributions and treatment responses, which are not captured in aggregate trends. Finally, although age-standardized rates were used for comparability, they may not fully account for evolving population age structures over the 32-year study period, potentially influencing both crude and standardized estimates (28).

An additional important limitation is that our study did not incorporate a formal sensitivity analysis. While the GBD estimates themselves undergo uncertainty quantification, we did not assess how variations in model assumptions, data inclusion criteria, or methodological choices might affect our specific comparative conclusions—particularly regarding age-stratified trends and SDI-related patterns. The absence of such analysis means that the stability and reliability of our reported associations, especially in regions with high modeling uncertainty, should be considered with appropriate caution (29).

## Conclusion

Based on our analysis of global data from 1990 to 2021, breast cancer (BC) prevalence increased among both women aged 20–54 years and those aged 55 years and older. In contrast, mortality and disability-adjusted life years (DALYs) exhibited divergent trends: these metrics decreased among older women but increased in the younger age group. Our regional comparisons further revealed that BC prevalence remains higher in high-SDI regions, although a declining trend has recently emerged. Concurrently, significant reductions in BC-related mortality and DALYs were observed in high- and upper-middle-SDI regions. These findings highlight the urgent need to implement effective interventions aimed at reducing the BC burden, particularly in lower-SDI regions and countries where the impact of the disease remains substantial.

## Data Availability

Publicly available datasets were analyzed in this study. This data can be found here: https://vizhub.healthdata.org/gbd-results/GBD Database.
